# Quality and impact of youtube for patient education on prostatic artery embolization

**DOI:** 10.1007/s00261-024-04790-y

**Published:** 2025-01-06

**Authors:** Connor C. Jacob, Mina S. Makary

**Affiliations:** The Ohio State University, Columbus, USA

**Keywords:** YouTube, Prostatic artery embolization, PAE, DISCERN

## Abstract

**Purpose:**

To evaluate the quality of YouTube videos on patient education concerning prostatic artery embolization (PAE).

**Methods:**

All PAE videos on YouTube were evaluated in December 2023. The quality of the videos was evaluated utilizing the DISCERN Scale Criterion. The popularity and engagement of each video was assessed using the Video Power Index (VPI) and Viewer Impact Score (VIS), respectively. Comparisons of these metrics were conducted and stratified by the video source type including academic institution, interventional radiologist, and patient testimony. Data describing discussion of risks, benefits, and indications were further collected.

**Results:**

Of the 43 videos, video characteristics included duration (mean = 4.6 min), views (mean = 16,885), and likes (mean = 139). The mean DISCERN, VPI, and VIS scores were 47.9, 15.0, and 36.9, respectively. There was no correlation between quality, and popularity (R^2^ = 0.09) or engagement (R^2^ = 0.01). Videos featuring board-certified physicians did not significantly improved DISCERN scores (*p* = 0.13), VPI (*p* = 0.15), or VIS (*p* = 0.39) scores when compared to those without. Content by interventional radiologists demonstrated higher popularity compared to videos featuring other specialties (*p* = 0.04), but there was no difference in quality (*p* = 0.18).

**Conclusion:**

Educational videos about PAE on YouTube are of average quality. Clinicians should be aware of the general state of online information concerning PAE and guide patients towards high quality resources.

## Introduction

Interventional radiology (IR) is a constantly evolving medical specialty that offers innovative and minimally-invasive procedures for a variety of conditions; however, much of IR is new and poorly understood by patients and providers [1, 2]. One such area is the treatment of benign prostatic hyperplasia (BPH). Common treatments for BPH refractory to medication include transurethral resection of the prostate, laser surgery, and the UroLift system. Prostatic artery embolization (PAE), a relatively recent minimally-invasive procedure performed by IR, was newly recognized in the American Urological Association (AUA) Guidelines for management of BPH in 2023 [3]. PAE is a safe therapy that is as effective as traditional prostatic surgeries for BPH treatment, but affords advantages such as reduced risk of bleeding and sexual dysfunction [4, 5].

Social media and online video libraries remain important sources for self-education on medical and procedural therapies. Over 2.5 billion users spent a cumulative one billion hours per day on YouTube as of 2020; however, YouTube has been shown to be a poorly reliable source of information on medicine and health [6, 7]. For instance, a study examining online resources for IR procedures, such as uterine fibroid embolization, determined online videos to be a low-to-mid quality source of information for patients [8]. Nonetheless, minimally-invasive procedures are often preferred by patients who, when surveyed, suggested short educational videos as effective ways to promote awareness of IR procedures [2]. To our knowledge, an evaluation of YouTube videos concerning PAE has never been published, and analysis of this material is important in understanding what exactly patients are exposed to when making decisions on BPH treatments, of which there are several. Reliable online information about IR procedures is critical in the context of low IR awareness among the public and increased internet utilization by patients. Therefore, the purpose of this study was to evaluate the characteristics and level of quality, popularity, and patient engagement of YouTube videos on PAE.

## Materials and methods

### Study design

This online-based study was performed with Institutional Review Board (IRB) exemption between August 12th and December 20th in 2023. This study did not require ethical approval because it solely utilized publicly available videos on YouTube without direct interaction with or intervention on human or animal subjects. All published educational videos about prostatic artery embolization on YouTube were evaluated using search criteria “prostatic artery embolization” OR “prostate artery embolization” OR “PAE.” Filters, such view count and relevance, were not utilized. Excluded videos were those that did not discuss PAE, did not provide educational content on PAE, or were business advertisements without educational intent on PAE. Duplicates were removed via evaluation of individual URLs for redundancy. An associate professor and practicing interventional radiologist with nearly a decade of performing PAE and a postgraduate medical student performed video selection and analysis.

### Video quality analysis

Descriptive statistics were collected for subcategories including videos delivered by interventional radiologists, non-IR physicians, non-physicians, and different institution types such as academic hospitals and medical device companies. These parameters include duration, views, likes, dislikes, comments, and days since upload (Table 1).

**Table 1:** Descriptive variables for PAE YouTube Videos

	Days Since Upload	Views	Duration (minutes)	Comments	Likes	Dislikes
Mean ± SD	1191 **±** 819	16,885 **±** 32,136	5 **±** 7	17 **±** 43	140 **±** 312	4 **±** 11
Minimum	105	84	1	0	0	0
Maximum	3371	179,811	44	252	1900	68

Standard deviation of the mean is abbreviated as “SD.”

The popularity of each video was assessed using the Video Power Index (VPI) [8, 9], and the level of patient engagement was measured using the Viewer Impact Score (VIS). VPI was calculated by the formula [(view ratio*like ratio) / 100)] [8, 10]. View ratios were calculated by dividing views by days since upload, and like ratios were calculated as follows: [(likes) / (likes/dislikes) * 100]. VIS aimed to evaluate positive patient engagement without potential skewing by very high or very low view counts, was calculated as [(comments + likes – dislikes) / video duration in minutes]. Finally, the quality of the videos was evaluated utilizing the DISCERN Scale Criteria [11]. The DISCERN Scale is battery of 15 questions tailored towards evaluating the suitability of health-centered information for patients and the public (Table 2). Each DISCERN criterion is graded on a Likert scale from 1 to 5 with a score of 1 representing “no,” 3 representing “partially,” and 5 representing “yes.” DISCERN scores between 63 and 72 are considered excellent, 51 to 62 as good, 39 to 50 as average, 28 to 38 as “poor” and < 28 as very poor [8, 10, 11].

**Table 2:** The 15-Question DISCERN scale criteria

Are the aims clear?	Does it provide details of additional sources of support and information?
Is it relevant?	Does it refer to areas of uncertainty?
Is it clear what sources of information were used to compile the publication?	Does it describe what would happen if no treatment was used?
Is it clear when the information used or reported in the publication was produced?	Does it describe how each treatment works?
Is it balanced and unbiased?	Does it describe the benefits of each treatment?
Does it achieve its aims?	Does it describe how the treatment choices affect overall quality of life?
Is it clear that there may be more than one possible treatment choice?	Does it provide support for shared decision making?
Does it describe the risks of each treatment?	

*Each of the fifteen DISCERN Scale questions are graded on a Likert scale from 1 to 5 for each video

Discussion of indications, contraindications, risk, benefits, alternative treatments, patient concerns, and recovery expectations were recorded for each video. The inclusion of patient testimonials was recorded, and the tone of each video was assessed for overall positivity, neutrality, or negativity towards PAE. Videos featuring positive comparative and superlative adjectives, upbeat music, appreciative patient testimonials, and overt treatment recommendation were considered to have a “positive” tone. Those delivered in an academic manner, without positive or negative descriptors, without spoken or written word, or with only diagrams or 3D renderings of the procedure were considered “neutral.” Videos with disparaging or cautious descriptions, overt recommendation against the procedure, or testimonials describing negative effects were considered “negative.”

### Statistical methods

Student t-tests and regression analyses were performed to compare the differences between categories and between videos with different characteristics and content. Comparative statistics analyzing differences in quality, popularity, engagement between author types were conducted. JMP 11 (SAS Institute Inc, Cary, NC) was used to assess all outcomes with the significance level set a priori at 0.05 for all statistical tests.

## Results

### Descriptive and demographic results

Forty-three YouTube videos were viewed in their entirety and analyzed using descriptive and quantitative measurements after exclusion of duplicates and videos without PAE for BPH treatment as the primary topic covered. Mean views per video was 16,885, mean video duration was 4.7 min, and mean like count was 140. Among all videos, there was a cumulative total of 720,071 views. Several types of organizations uploaded videos including private medical practices (47%), academic hospitals (40%), medical device companies (7%), and educational websites (7%). 31 videos were delivered by board-certified physicians of which 26 were interventional radiologists. Videos were assessed for discussion of indications (95%), contraindications (9%), benefits (91%), risks (35%), alternatives (47%), patient concerns (56%), and recovery expectations (74%). Nine videos were delivered with a neutral tone (21%) and thirty-four with a positive tone (79%).

### DISCERN, VPI, and VIS

For all videos included in the study, mean DISCERN, VPI, and VIS scores were 47.9, 15.0, and 36.9, respectively (Table 3). No correlation was found between quality and popularity (R^2^ = 0.09) or engagement (R^2^ = 0.01) for all videos. There was moderate correlation between popularity and engagement (R^2^ = 0.53). Videos featuring board-certified physicians did not have significantly different DISCERN (*p* = 0.13), VPI (*p* = 0.15), or VIS (*p* = 0.39) scores when compared to non-physician videos. Interventional radiologists were found to have significantly higher popularity compared to videos featuring urologists or a medical oncologist (*p* = 0.04), but no difference was found in quality (*p* = 0.18) or engagement (*p* = 0.14). The inclusion of patient testimonials had no effect on quality (*p* = 0.79), popularity (*p* = 0.76), or engagement (*p* = 0.84). Engagement with videos published by academic hospitals was higher than private hospitals (*p* = 0.02). There were four videos, without a common author type, evaluated as having poor quality with DISCERN scores less than 38. Three videos were found to have “excellent” quality; each of these was delivered by an interventional radiologist. Videos featuring speech and text demonstrated an average DISCERN of 50.7 while videos featuring only speech, only text, or no speech or text had an average DISCERN of 42.39 (*p* = 0.03). There was no difference in popularity between these subgroups *p* = 0.11).

**Table 3:** DISCERN, VPI, and VIS Data for Physicians, Specialties, non-physicians, and institutions

	DISCERN (Mean ± SD)	VPI (Mean ± SD)	VIS (Mean ± SD)
**All** (*n* = 43)	47.88 ± 8.3	15.00 ± 22.9	36.89 ± 55.6
**Physician Categories**			
**Physicians** (*n* = 31)	**49.19 ± 7.8**	11.54 ± 21.5	30.39 ± 19.1
**IR** (*n* = 26)	49.89 **±** 8.1	**13.19 ± 23.2**	33.09 **±** 39.6
**Non-IR Physicians** (*n* = 5)	45.60 **±** 5.3	**2.97 ± 3.2**	16.33 **±** 16.4
**Non-Physicians** (*n* = 12)	44.50 **±** 8.9	23.93 **±** 30.9	**53.70 ± 86.6**
**Institutional Categories**			
**Academic Hospitals** (*n* = 17)	**50.88 ± 8.5**	13.85 **±** 27.9	31.73 **±** 44.6
**Private Hospitals** (*n* = 3)	46.33 **±** 0.6	2.76 **±** 1.6	3.74 **±** 3.2
**Private Practices** (*n* = 17)	47.06 **±** 8.4	16.09 **±** 19.4	36.42 **±** 33.9
**Medical Device Companies** (*n* = 3)	42.67 **±** 12.1	21.92 **±** 26.8	**101.43 ± 175.7**
**Educational/Research Websites** (*n* = 3)	44.00 **±** 6.6	20.65 **±** 26.2	11.67 **±** 28.4

This table describes DISCERN, VPI, and VIS scores for video categories and subcategories. The “Physicians” category includes all videos delivered by physicians (interventional radiologists, urologists, and a medical oncologist). The “Non-IR Physicians” subcategory is composed of videos created by urologists and a medical oncologist. The “Non-Physicians” category includes all videos delivered by non-board-certified spokespersons

DISCERN criterion demonstrating less than a 3/5 (“partial” discussion of a given topic) across all videos were citing of source material (2.6), publication date of source material (2.7), additional sources of information (1.4), reference to areas of uncertainty (1.3), discussion of expected outcomes with no treatment (2.6), discussion of risks (1.9), and presentation of alternative treatments (2.65). Additionally, five videos had significantly higher popularity (*p* = 0.02) without differences in quality (*p* = 0.25) or engagement (*p* = 0.06) compared to the rest of the dataset. These videos garnered a far higher average view (80,407) and like counts (741) in comparison to the rest of the data set. They discussed procedural indications (100%), contraindications (20%), benefits (80%), risks (40%), alternatives (60%), patient concerns (60%), and recovery (60%) at roughly the same rate as the rest of the dataset. Four of these videos were delivered with a positive tone and one with a neutral tone.

## Discussion

BPH is a frustrating diagnosis for millions of men in the United States with sharply increasing incidence in the later decades of life [12]. PAE is a safe and effective minimally-invasive intervention for BPH that was recently recognized by the AUA; however, awareness of this procedure and the specialty of IR, in general, is low among primary care practitioners and in the public [1, 2]. The internet affords patients with the opportunity to explore treatment options such as PAE, and online video platforms such as YouTube serve a prominent role in this form of self-education. Indeed, patients often come to physicians with preexisting knowledge of medical interventions because of websites like YouTube, but sometimes fail to distinguish reliable from unreliable material [13]. To evaluate YouTube videos on PAE, our primary outcomes of interest were quality, popularity, and engagement. Our analyses revealed that these videos demonstrate average quality as determined by the DISCERN scale criterion. This result indicates that PAE videos have higher average quality than videos on uterine fibroid embolization, meningiomas, men’s health, and patellofemoral instability, to reference other studies of a similar type [8, 14–16]. No correlation was found between quality and popularity or engagement, but there was a positive correlation between popularity and engagement.

The first outcome of interest, DISCERN, can be used to evaluate individual criteria of quality such as “Is it [the video] balanced and unbiased?” or “Does it describe how each treatment works?” DISCERN scores less than three are generally considered to be “low,” while scores greater than 3 are considered to be adequate for each specific criterion [11]. Most videos centered around positive marketing without significant discussion of downsides of procedure; indeed, this study determined that DISCERN criteria among all videos were notably lower in areas of uncertainty, sources of information for further patient education, and risks. DISCERN areas with the highest scores were presentation of clear aims, relevance, achievement of aims, description of benefits of treatment, and support for shared decision making.

As demonstrated in published literature, DISCERN can also be used to assess the quality of information presented by a video as a summative score of each criterion [8, 10, 11]. Each selected subgroup, whether institution type, physician, non-physician, or physician specialty, was found to have average levels of quality according to this summative scoring method. The three videos with the highest DISCERN scores were published by IR; these videos approached PAE discussion comprehensively and featured more extensive conversation about procedural details and patient expectations post-operatively. Indications, benefits, patient concerns, and recovery expectations were discussed by most videos, while risks, alternative treatments, and patient concerns were discussed by less than half. This result wasn’t unexpected especially when considering that most videos recommended the procedure and were delivered with a positive tone. Based on these results, future content creators interested in producing educational videos on PAE and similar topics could definitively improve video quality by clearly describing risks, alternatives, and areas of uncertainty and by citing source material. Furthermore, multiple modalities of information presentation including speech accompanied by text was shown to improve quality. The selected quality metrics may favor videos with both speech and text because they have the potential to cover more content while maintaining readability with simple bulleted slides. Other potential beneficial formats including animation, large-text bullet take-aways, and high-resolution video of the speaker may enhance educational value for patients, especially those requiring accessibility accommodations. Interestingly, the increased DISCERN values observed in the combined speech and text videos was not accompanied by a statistically significant increase in popularity for multimodality presentation styles.

VPI scoring describes popularity as a function of likes and views with a minimum of zero, in the case of zero likes, and an unrestricted maximum dependent on like and view count. PAE videos had lower VPI scores (mean = 15.0) compared to videos on uterine fibroid embolization (mean = 66.4), kyphosis (mean = 174.4), and labral tears (mean = 33.53) [8, 9, 17]. PAE videos delivered by board-certified IR had higher popularity when compared to other specialties. This finding should be interpreted in the context of relatively low public awareness of the IR and suggests that patients did not actively avoid IR videos, though there is no evidence that patients actively sought these videos out [2]. Videos delivered by urologists, and one by a medical oncologist, had significantly lower view counts, which is the most important factor determining a video’s popularity. IR did not demonstrate increased popularity when compared to non-board-certified physician sources, which had higher average view counts. An explanation for this disparity in views is not immediately apparent, but may be due to discrepancies in marketing strategies, the order of search results according to relevance, and shorter video duration.

Engagement, quantified by VIS, aimed to assess the level of positive user interaction with the video in a time-bound fashion according to number of comments, likes, and dislikes per minute. A VIS score of zero can occur in the absence of likes, comments, and dislikes, or when the number of dislikes is equal to the combined number of comments and likes. VIS has no defined upper threshold because YouTube does not cap comments or likes. Engagement and popularity were found to be correlated. This correlation is likely explained by the fact that formulas for both VIS and VPI take likes and dislikes into account; while VPI also incorporates views, videos with more views also received more likes. Viewers did not demonstrate preferential engagement for videos published by IR, urologists, or non-boarded spokespeople. It was observed that viewers demonstrated marked engagement with videos published by academic hospitals in comparison to private hospitals. This could be because accounts of academic hospitals with name recognition included in this study, such as Johns Hopkins, Mount Sinai, and Stanford medical centers, had more views when compared to private hospital accounts.

In addition, further comparison of the five most popular videos was made to the rest of the dataset to evaluate more deeply the relationship between popularity and quality. These videos were delivered by various sources including an IR from an academic institution and representatives of a medical device company, an educational website, and two private practices. While these five videos were significantly more popular in terms of the number of views, they did not exhibit higher quality as measured by DISCERN. This finding supports the conclusions of existing literature on YouTube’s lack of consistency in education and dissemination of high-quality information to patients, and that accredited institutional accounts are sometimes not any more popular than other account types [10, 18]. One such study examining the state of information concerning palliative care found that advocacy organizations had several times more popularity than health care groups [19]. However, it should be noted that YouTube has been found to have higher quality medical information than other forms of social media like Twitter and Instagram [20].

Limitations of this study include the sample size which did not capture every video on PAE accessible to patients. However, all the relevant and most viewed videos on YouTube consistent with our search strategy were included, and in total, the selected videos represent a comprehensive sample of the different characteristics and publishing author types available to the public. Additionally, video authors generally presented information with a positive tone and did not discuss risks, implying potential creator bias throughout the collection of included videos. This study was also limited by a single author conducting DISCERN scoring, which is a subjective rubric, but multiple studies have demonstrated near perfect alignment between graders when using the DISCERN criteria [8, 10, 15]. Future studies would benefit from multi-grader scoring. Moreover, our study did not include analysis of search engine optimization and search relevance strategies carried out by individual accounts. The literature would benefit from analysis of these factors in future studies.

In conclusion, YouTube videos on PAE demonstrated an average level of quality according to DISCERN. Interventional radiologist videos were more popular than videos created by other medical specialties. No relationship was found between popularity and quality even when examining the most popular and highly viewed videos in comparison to the rest of the dataset. Further examination of the quality and reliability of online information about IR procedures is especially important given the public’s low awareness of the field.

## Data Availability

No datasets were generated or analysed during the current study.
